# Vertebral Artery Dissection: A Pain in the Neck

**DOI:** 10.7759/cureus.12985

**Published:** 2021-01-29

**Authors:** Brian R Covello, Anjeza Chukus

**Affiliations:** 1 Radiology, Aventura Hospital and Medical Center, Aventura, USA

**Keywords:** vertebral artery dissection, stroke

## Abstract

Vertebral artery dissection (VAD) is increasingly identified as a cause of ischemic stroke in young adults. Patients most commonly present with neck pain, headache, visual disturbance, or focal extremity weakness. We present a case of spontaneous VAD in a patient whose only symptoms at presentation were neck pain and headache.

A 42-year-old male presented to the emergency department with one week of left neck pain and headache. Computed tomography (CT) neck with contrast was initially ordered for neck pain. CT neck revealed an incidental anterior communicating artery (ACOM) aneurysm. Digital subtraction angiography (DSA) performed for ACOM aneurysm coiling demonstrated a left VAD, which was the attributable etiology to the patient's presentation. Subsequent magnetic resonance angiogram (MRA) neck confirmed this finding. Follow-up brain MRI revealed a small acute left occipital lobe infarct secondary to thromboembolism from the VAD. The patient underwent endovascular coiling of the ACOM aneurysm and received aspirin for the VAD, obtaining resolution of his symptoms.

VAD involves an intimal tear of the vasa vasorum leading to narrowing of the vessel lumen that can result in thromboembolic complications. Risk factors for development of VAD include neck manipulations, trauma, or abnormal posturing. DSA remains the gold standard imaging exam for diagnosis of VAD. However, recognition of VAD on more common non-invasive modalities, such as computed tomography angiogram or MRA, remains critical for establishing the correct diagnosis. Although the clinical presentation of VAD is highly variable, dissection should be considered in a young patient with craniocervical pain, even in the absence of neurological symptoms. Early diagnosis and treatment of VAD can lower the risk of long-term neurologic sequelae.

## Introduction

Vertebral artery dissection (VAD) accounts for less than 2.5% of all strokes and disproportionately affects younger, healthier patients [1,2]. VAD involves an intimal tear through the vasa vasorum, leading to localized hemorrhage, narrowing of the vessel lumen, and thromboembolic complications [3]. Etiologies include neck manipulations, trauma, abnormal posturing, or connective tissue disorders, yet studies have suggested that these risk factors are not identified in a majority of individuals [4,5]. Digital subtraction angiography (DSA) is the gold standard exam for the diagnosis of VAD; however, detection of this abnormality is increasingly identified on the more commonly used non-invasive imaging modalities, such as computed tomography angiogram (CTA) or magnetic resonance angiogram (MRA).

Herein, we present a case of spontaneous VAD in a patient whose only symptoms at presentation were neck pain and headache.

## Case presentation

**Figure 1:**
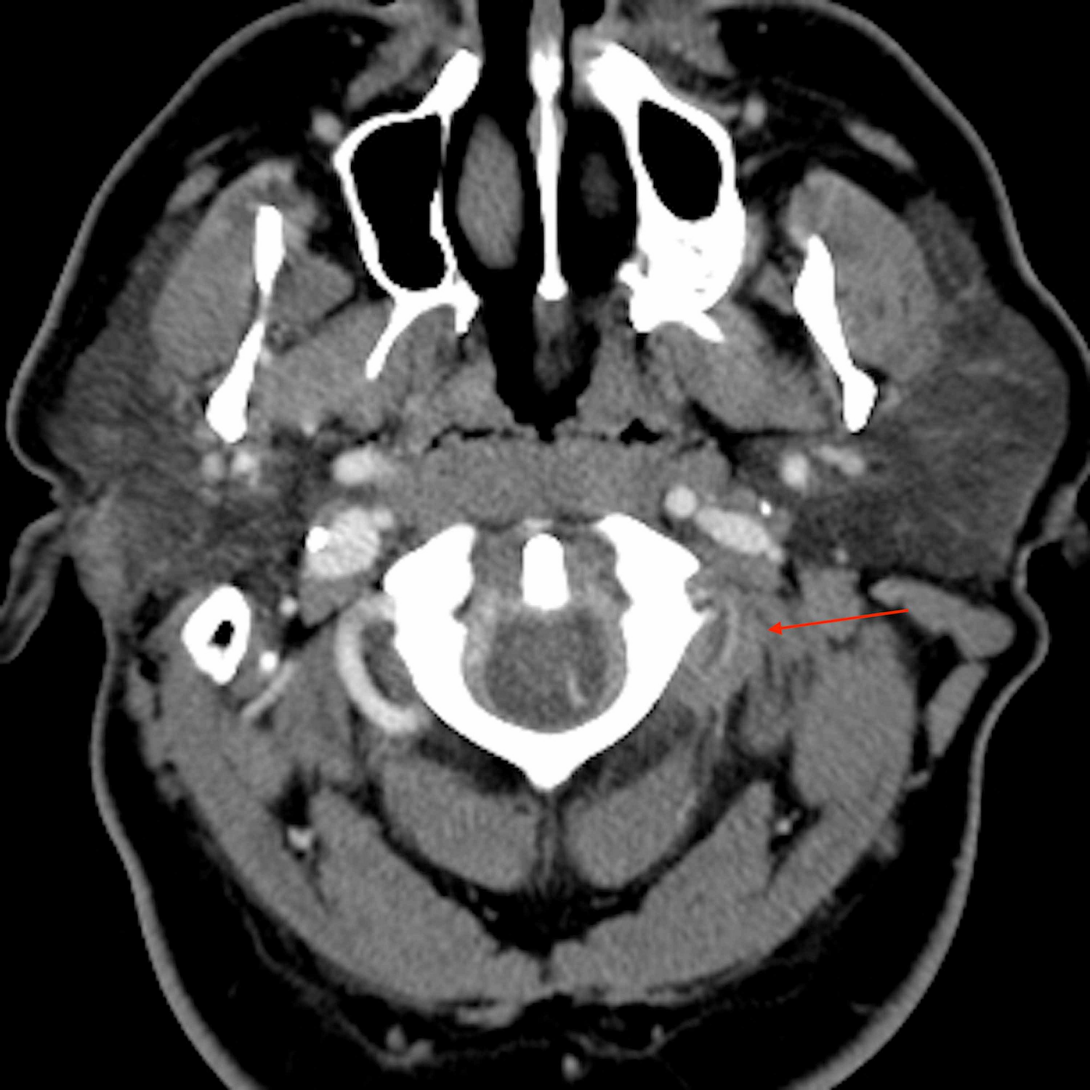
Axial-view contrast-enhanced neck CT performed as the initial imaging exam shows narrowed V3 segment with surrounding isodense hematoma.

A 42-year-old male patient with past medical history of hypertension presented to our institution with one week of worsening headache and neck pain. He denied focal neurological deficits. CT neck with contrast was initially ordered for neck pain. CT neck revealed an incidental 0.5-cm ACOM aneurysm, and a dissection involving the third segment (V3) of the left vertebral artery was noted in retrospect after DSA (Figure 1). 

**Figure 2:**
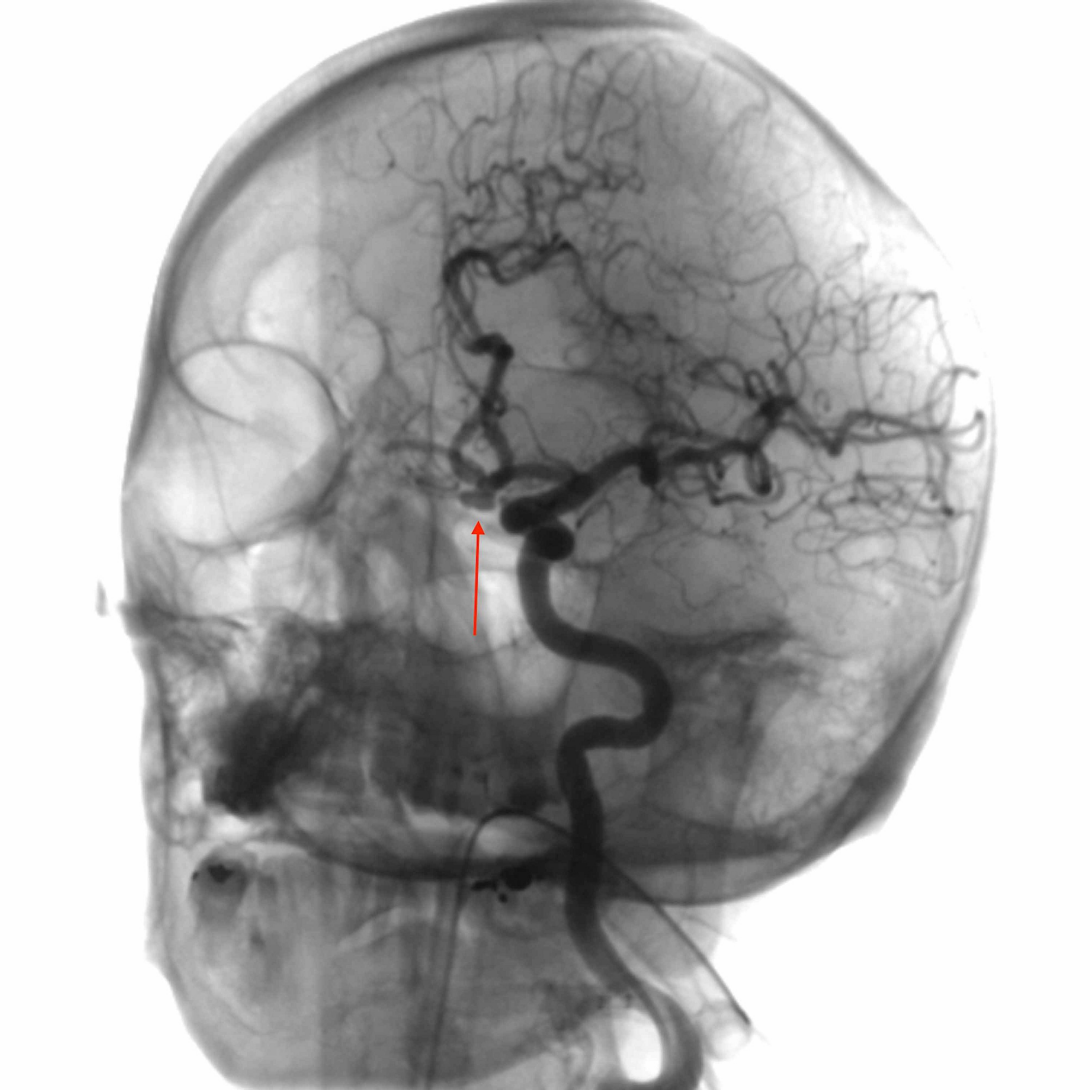
Oblique-view digital subtraction angiography with left vertebral artery selection reveals a 0.5-cm anterior communicating artery aneurysm.

**Figure 3:**
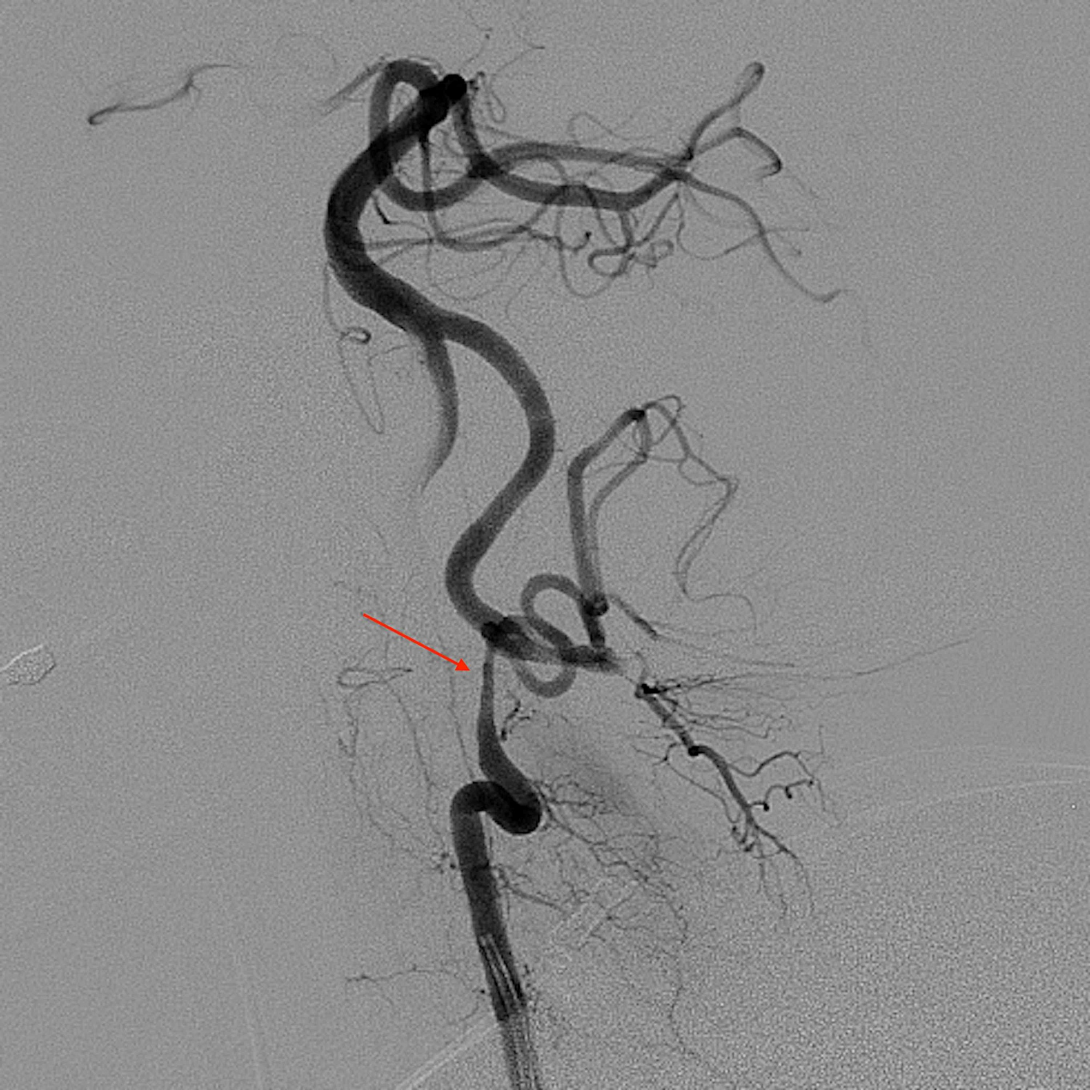
Oblique-view digital subtraction angiography with left vertebral artery selection shows vessel wall irregularity with tapering and abrupt marked narrowing of the V3 segment of the left vertebral artery.

The DSA performed by the neurointerventional radiologist for ACOM aneurysm coiling revealed moderate narrowing and vessel wall irregularity of the left vertebral artery V3 segment compatible with VAD (Figure 2, Figure 3). In retrospect, this finding was identifiable on the initial CT neck examination and was found to be the etiology of the patient’s symptoms at presentation. The patient successfully underwent coiling of the ACOM aneurysm and was started on aspirin monotherapy for the VAD.

**Figure 4:**
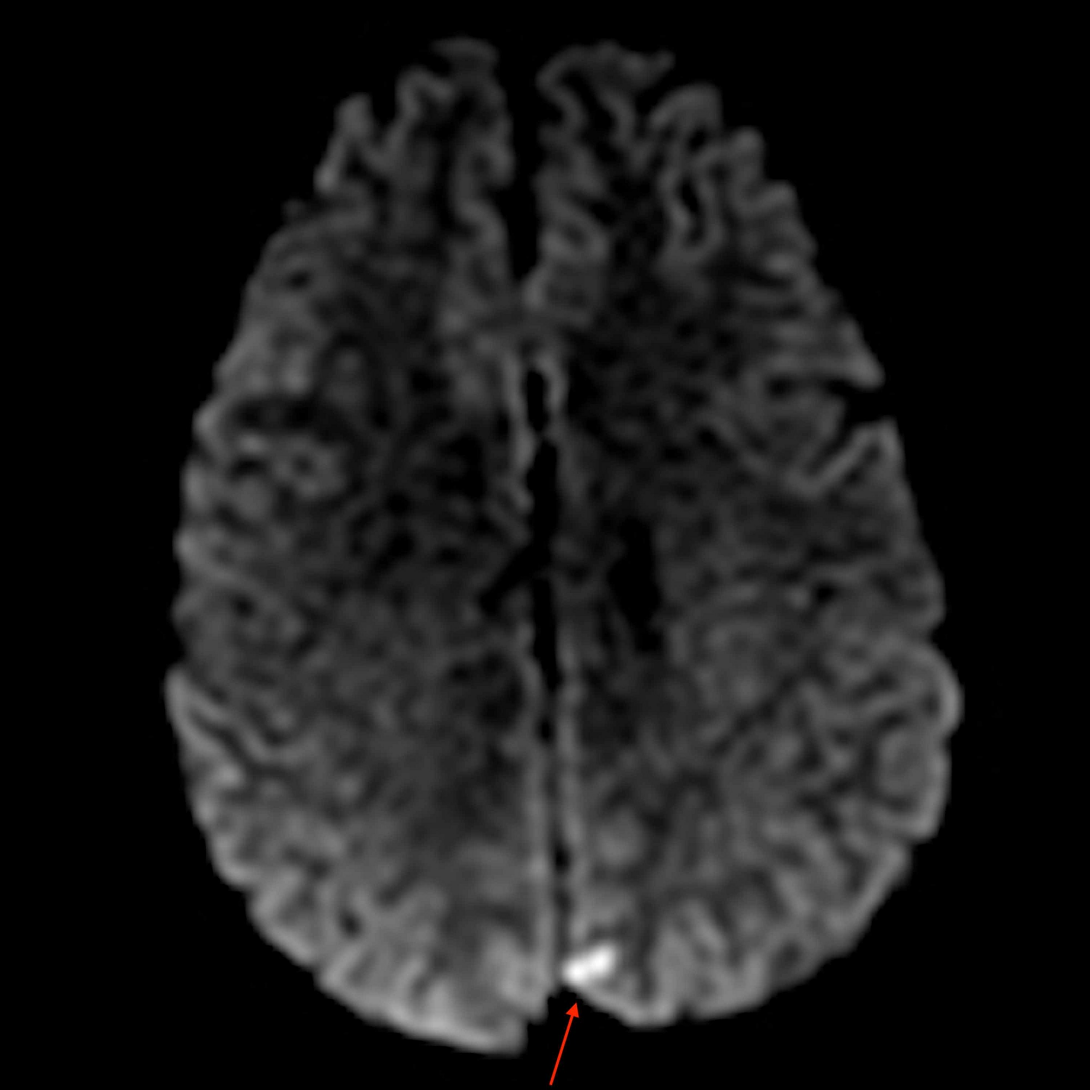
Diffusion-weighted MRI shows a small focus of diffusion restriction in the left occipital cortex compatible with an acute small vessel embolic infarct.

**Figure 5:**
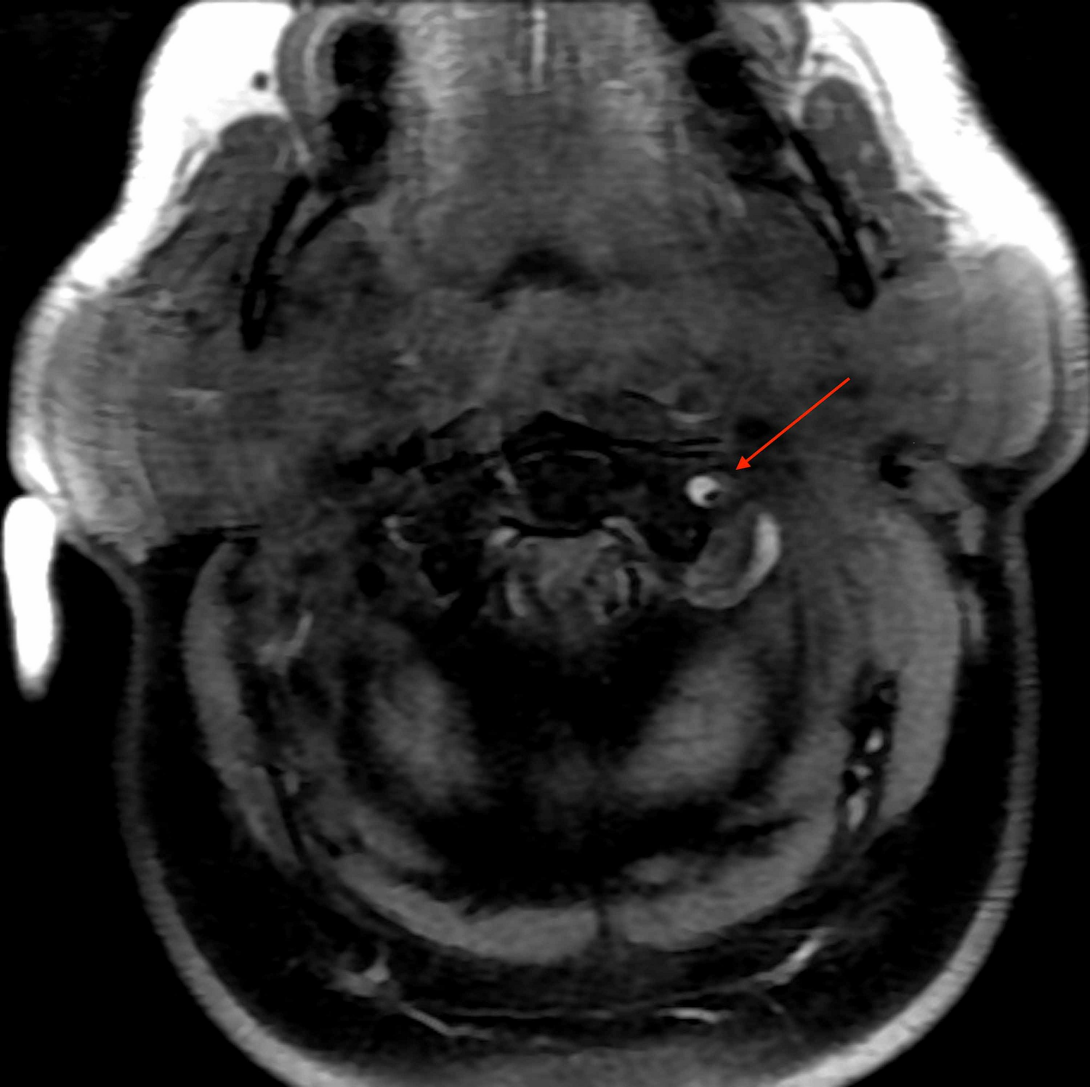
Axial T1 MRI at the C1-C2 level demonstrates T1 hyperintense crescent-shaped false lumen with T1 hypointense narrowed true lumen.

A post-DSA brain MRI showed a small focus of diffusion restriction in the left occipital cortex with associated T2 and fluid-attenuated inversion recovery hyperintense signal, consistent with a late acute infarct secondary to thromboembolic complications from the left VAD (Figure 4). Follow-up MRA neck also demonstrated the VAD with a T1 hyperintense crescent-shaped false lumen with a constricted and irregular true lumen at the V3 left vertebral artery segment (Figure 5). The patient had complete resolution of his symptoms prior to discharge.

## Discussion

VAD-related ischemic events disproportionately affect a younger, healthier population compared to other stroke subtypes, with one study showing a mean age of 44.1 years at diagnosis [4]. Patients with VAD most commonly present with vague and non-specific symptoms of headache and neck pain [6]. Patients may also present with neurologic symptoms related to posterior circulation ischemia [7]. Given the non-specificity and variability of clinical presentation, diagnosis of VAD is oftentimes difficult, and a high degree of suspicion is needed to make a clinical diagnosis. Angiography is the gold standard for the diagnosis of VAD on imaging. Increasing utilization of non-invasive imaging in recent decades has allowed more patients to be diagnosed with VAD [8]. One study showed that cervical artery dissections are most commonly identified on MRA, followed by conventional angiography and CTA [4].

While headache and neck pain are very common medical complaints of patients in the emergency department, and usually of benign etiology, they may represent early symptoms of VAD. Our patient’s symptoms at presentation were neck pain and headache, but the VAD was missed on the initial neck CT with contrast, and subsequent work-up of an incidental ACOM aneurysm ultimately led to the correct diagnosis. In hindsight, CT neck with contrast demonstrated the left V3 segment vertebral artery luminal irregularity and narrowing.

When a patient is suspected of having VAD, CTA neck or MRA neck with T1 fat-suppressed sequence should be the next appropriate test. Contrast-enhanced MRA is sensitive but not specific for VAD unless combined with axial T1 fat-suppressed sequence [9]. The MRA neck with T1 fat-suppressed sequence would typically demonstrate the classical finding of crescent-shaped T1 hyperintense false lumen, suggesting an intramural hematoma [1].

Pathognomonic findings of VAD on DSA include identification of an intimal flap or double lumen; however, these findings are identified in less than 10% of cases [4]. Most commonly, DSA will reveal sudden tapering secondary to luminal irregularity, such as in our patient [4,10].

VADs are divided into intracranial and extracranial subtypes, and have been classified into four segments, V1 through V4. The V3 segment is located in the region of the cervical spine with the greatest range of motion (C1-C2 level) and is predisposed to motion-related injuries [3,7]. Dissection location determines prognosis and guides management. Intracranial V4 segment extension is associated with poorer outcomes due to its association with subarachnoid hemorrhage [10]. While extracranial VADs are typically treated with anti-platelet therapy, this therapy is contraindicated in intracranial VAD due to the increased risk of subarachnoid hemorrhage [3].

VAD can be a nidus for future thromboembolic events [10]. As such, it represents a treatable cause of stroke. Early recognition of VAD is critical, as progression to stroke can be avoided by appropriate and timely medical therapy [11]. Important clinical symptoms that suggest dissection include any focal neurologic deficit in the setting of neck pain or headache. When dissection is suspected, CTA neck would be the next appropriate test. A high degree of clinical suspicion in combination with early detection on imaging is critical to a prompt diagnosis. Although DSA remains the gold standard, identification of this entity on more commonly utilized non-invasive imaging modalities may help prevent devastating neurological consequences with the appropriate therapy.

## Conclusions

VADs account for a minority of ischemic strokes in the general population; however, they represent a greater proportion of ischemic strokes in young, relatively healthy patients. VAD should be included in the differential diagnosis of young patients with craniocervical pain, even in the absence of neurological deficits. Radiological identification of VAD on non-invasive imaging modalities can help ensure early detection of this otherwise clinically difficult diagnosis. 
